# Supplementary material for: Multi‐dataset identification of innovative feature genes and molecular mechanisms in keratoconus
Source: J Cell Mol Med. 2024 Sep 19;28(18):e70079. doi: 10.1111/jcmm.70079 (PMC11412914; doi:10.1111/jcmm.70079)
Supplement: Supplementary file 2 — Material S2. [file JCMM-28-e70079-s002.zip › 7.GSEA analysis/2.ATOH7/KEGG_OOCYTE_MEIOSIS.html]

Details for gene set KEGG\_OOCYTE\_MEIOSIS[GSEA]

|  || Dataset | ATOH7.group.cls#h\_versus\_l |
| Phenotype | group.cls#h\_versus\_l |
| Upregulated in class | l |
| GeneSet | KEGG\_OOCYTE\_MEIOSIS |
| Enrichment Score (ES) | -0.14723113 |
| Normalized Enrichment Score (NES) | -0.75071496 |
| Nominal p-value | 0.81636727 |
| FDR q-value | 1.0 |
| FWER p-Value | 1.0 |
Table: GSEA Results Summary

  

Fig 1: Enrichment plot: KEGG\_OOCYTE\_MEIOSIS      
 Profile of the Running ES Score & Positions of GeneSet Members on the Rank Ordered List

  

| SYMBOL | TITLE | RANK IN GENE LIST | RANK METRIC SCORE | RUNNING ES | CORE ENRICHMENT || 1 | PRKACB | na | 829 | 0.474 | 0.0200 | No |
| 2 | PRKACG | na | 1784 | 0.361 | 0.0259 | No |
| 3 | PLK1 | na | 1965 | 0.347 | 0.0529 | No |
| 4 | CAMK2D | na | 2442 | 0.313 | 0.0682 | No |
| 5 | CALML6 | na | 3497 | 0.256 | 0.0616 | No |
| 6 | MAP2K1 | na | 4240 | 0.228 | 0.0612 | No |
| 7 | YWHAH | na | 4637 | 0.213 | 0.0696 | No |
| 8 | CPEB1 | na | 5079 | 0.199 | 0.0753 | No |
| 9 | ADCY3 | na | 5082 | 0.198 | 0.0936 | No |
| 10 | RPS6KA1 | na | 5757 | 0.178 | 0.0907 | No |
| 11 | CDC25C | na | 6171 | 0.166 | 0.0941 | No |
| 12 | MAPK12 | na | 6613 | 0.154 | 0.0956 | No |
| 13 | BUB1 | na | 7052 | 0.142 | 0.0962 | No |
| 14 | PPP3CC | na | 7238 | 0.137 | 0.1035 | No |
| 15 | ITPR1 | na | 7473 | 0.131 | 0.1090 | No |
| 16 | SMC3 | na | 8427 | 0.110 | 0.0916 | No |
| 17 | FBXO5 | na | 8435 | 0.109 | 0.1015 | No |
| 18 | PPP2CB | na | 8608 | 0.106 | 0.1064 | No |
| 19 | SPDYA | na | 8647 | 0.106 | 0.1151 | No |
| 20 | MAD2L2 | na | 8719 | 0.104 | 0.1227 | No |
| 21 | PPP2CA | na | 9540 | 0.090 | 0.1073 | No |
| 22 | CALM3 | na | 10070 | 0.080 | 0.0995 | No |
| 23 | ADCY8 | na | 10448 | 0.075 | 0.0955 | No |
| 24 | CDC26 | na | 10763 | 0.070 | 0.0929 | No |
| 25 | RPS6KA6 | na | 10771 | 0.070 | 0.0992 | No |
| 26 | PPP2R5B | na | 11069 | 0.065 | 0.0966 | No |
| 27 | INS | na | 11499 | 0.059 | 0.0897 | No |
| 28 | PPP3CA | na | 11626 | 0.057 | 0.0914 | No |
| 29 | PPP3CB | na | 11848 | 0.055 | 0.0901 | No |
| 30 | CCNB2 | na | 12045 | 0.052 | 0.0892 | No |
| 31 | YWHAQ | na | 12136 | 0.051 | 0.0913 | No |
| 32 | PPP2R1A | na | 12268 | 0.049 | 0.0921 | No |
| 33 | PRKACA | na | 12347 | 0.048 | 0.0943 | No |
| 34 | SPDYC | na | 12381 | 0.048 | 0.0978 | No |
| 35 | PLCZ1 | na | 12549 | 0.045 | 0.0972 | No |
| 36 | REC8 | na | 12606 | 0.045 | 0.0997 | No |
| 37 | CDC27 | na | 12677 | 0.044 | 0.1017 | No |
| 38 | PRKX | na | 12697 | 0.044 | 0.1052 | No |
| 39 | PPP2R5C | na | 12732 | 0.043 | 0.1083 | No |
| 40 | ANAPC10 | na | 12863 | 0.042 | 0.1084 | No |
| 41 | PKMYT1 | na | 12892 | 0.042 | 0.1115 | No |
| 42 | CCNB1 | na | 13128 | 0.039 | 0.1083 | No |
| 43 | CDC20 | na | 13189 | 0.038 | 0.1101 | No |
| 44 | MOS | na | 13525 | 0.035 | 0.1037 | No |
| 45 | PPP1CC | na | 14047 | 0.030 | 0.0914 | No |
| 46 | MAPK1 | na | 14536 | 0.025 | 0.0797 | No |
| 47 | CHP1 | na | 14674 | 0.024 | 0.0780 | No |
| 48 | SKP1 | na | 14712 | 0.024 | 0.0791 | No |
| 49 | PPP3R1 | na | 15411 | 0.018 | 0.0605 | No |
| 50 | PPP2R5E | na | 15532 | 0.017 | 0.0586 | No |
| 51 | YWHAG | na | 16222 | 0.011 | 0.0397 | No |
| 52 | CALM1 | na | 16251 | 0.011 | 0.0399 | No |
| 53 | ANAPC2 | na | 16554 | 0.008 | 0.0320 | No |
| 54 | MAPK3 | na | 16821 | 0.006 | 0.0249 | No |
| 55 | PTTG1 | na | 16942 | 0.005 | 0.0219 | No |
| 56 | CUL1 | na | 17124 | 0.003 | 0.0169 | No |
| 57 | ANAPC11 | na | 17183 | 0.003 | 0.0155 | No |
| 58 | CDC16 | na | 17246 | 0.002 | 0.0139 | No |
| 59 | PPP1CA | na | 17263 | 0.002 | 0.0137 | No |
| 60 | BTRC | na | 17413 | 0.001 | 0.0094 | No |
| 61 | PPP2R5D | na | 17451 | 0.001 | 0.0084 | No |
| 62 | PTTG2 | na | 17575 | -0.000 | 0.0049 | No |
| 63 | MAD2L1 | na | 17730 | -0.002 | 0.0006 | No |
| 64 | CDK2 | na | 18103 | -0.005 | -0.0097 | No |
| 65 | ANAPC5 | na | 18683 | -0.010 | -0.0255 | No |
| 66 | ITPR3 | na | 18764 | -0.011 | -0.0268 | No |
| 67 | SMC1A | na | 19364 | -0.016 | -0.0426 | No |
| 68 | PGR | na | 19564 | -0.018 | -0.0467 | No |
| 69 | AURKA | na | 19592 | -0.018 | -0.0458 | No |
| 70 | PPP1CB | na | 19627 | -0.018 | -0.0451 | No |
| 71 | SMC1B | na | 19661 | -0.019 | -0.0443 | No |
| 72 | YWHAB | na | 19781 | -0.020 | -0.0459 | No |
| 73 | FBXW11 | na | 19927 | -0.021 | -0.0482 | No |
| 74 | CDC23 | na | 20426 | -0.026 | -0.0601 | No |
| 75 | YWHAZ | na | 20490 | -0.027 | -0.0595 | No |
| 76 | CALML3 | na | 20596 | -0.028 | -0.0600 | No |
| 77 | ADCY9 | na | 20701 | -0.029 | -0.0603 | No |
| 78 | RPS6KA3 | na | 20863 | -0.030 | -0.0622 | No |
| 79 | ANAPC13 | na | 21067 | -0.032 | -0.0650 | No |
| 80 | ANAPC4 | na | 21182 | -0.034 | -0.0652 | No |
| 81 | SLK | na | 21547 | -0.038 | -0.0722 | No |
| 82 | ADCY2 | na | 21650 | -0.039 | -0.0716 | No |
| 83 | RBX1 | na | 21760 | -0.040 | -0.0710 | No |
| 84 | STAG3 | na | 21940 | -0.042 | -0.0722 | No |
| 85 | CALM2 | na | 21949 | -0.042 | -0.0685 | No |
| 86 | ANAPC7 | na | 22004 | -0.043 | -0.0661 | No |
| 87 | CAMK2A | na | 22272 | -0.046 | -0.0696 | No |
| 88 | CAMK2G | na | 23214 | -0.058 | -0.0914 | No |
| 89 | CCNE1 | na | 23221 | -0.058 | -0.0862 | No |
| 90 | PPP2R5A | na | 23614 | -0.064 | -0.0916 | No |
| 91 | AR | na | 25031 | -0.086 | -0.1245 | No |
| 92 | ADCY5 | na | 25230 | -0.089 | -0.1220 | No |
| 93 | ADCY6 | na | 25322 | -0.091 | -0.1162 | No |
| 94 | YWHAE | na | 25594 | -0.096 | -0.1151 | No |
| 95 | ADCY7 | na | 26708 | -0.119 | -0.1362 | Yes |
| 96 | ITPR2 | na | 26843 | -0.122 | -0.1288 | Yes |
| 97 | RPS6KA2 | na | 27234 | -0.132 | -0.1278 | Yes |
| 98 | ADCY4 | na | 27391 | -0.136 | -0.1197 | Yes |
| 99 | IGF1R | na | 27632 | -0.142 | -0.1135 | Yes |
| 100 | ESPL1 | na | 27791 | -0.145 | -0.1046 | Yes |
| 101 | CCNE2 | na | 28205 | -0.157 | -0.1020 | Yes |
| 102 | CHP2 | na | 28497 | -0.164 | -0.0952 | Yes |
| 103 | PPP2R1B | na | 28524 | -0.165 | -0.0807 | Yes |
| 104 | ANAPC1 | na | 29468 | -0.193 | -0.0900 | Yes |
| 105 | CDK1 | na | 29770 | -0.203 | -0.0799 | Yes |
| 106 | CALML5 | na | 29931 | -0.208 | -0.0652 | Yes |
| 107 | ADCY1 | na | 30908 | -0.244 | -0.0707 | Yes |
| 108 | FBXO43 | na | 31201 | -0.254 | -0.0556 | Yes |
| 109 | PPP3R2 | na | 32636 | -0.337 | -0.0657 | Yes |
| 110 | IGF1 | na | 33039 | -0.370 | -0.0430 | Yes |
| 111 | CAMK2B | na | 34693 | -0.989 | 0.0010 | Yes |
Table: GSEA details [plain text format]

  

Fig 2: KEGG\_OOCYTE\_MEIOSIS      
 Blue-Pink O' Gram in the Space of the Analyzed GeneSet

  

Fig 3: KEGG\_OOCYTE\_MEIOSIS: Random ES distribution      
 Gene set null distribution of ES for **KEGG\_OOCYTE\_MEIOSIS**
